# Psychological Well-being After Body Contouring Surgery: Hormonal and Surgical Predictors

**DOI:** 10.1007/s00266-026-05831-1

**Published:** 2026-04-13

**Authors:** Mohamed Badie Ahmed, Abeer Alsherawi, Salma Jarrar, Fatima Saoud Al-Mohannadi, Saif Badran, Asma Syed, Hoda Khoogaly, Suhail A. Doi, Abdella M. Habib

**Affiliations:** 1 Department of Population Medicine, College of Medicine, QU Health, Qatar University, Doha, Qatar; 2 Plastic Surgery Department, Hamad General Hospital, Hamad Medical Corporation, Doha, Qatar; 3 College of Medicine, QU Health, Qatar University, Doha, Qatar; 4 Division of Plastic and Reconstructive Surgery, Washington University School of Medicine, Saint Louis, MO USA; 5 Qatar Metabolic Institute, Academic Health System, Hamad Medical Corporation, PO Box 3050, Doha, Qatar; 6 Department of Basic Medical Sciences, College of Medicine, QU Health, Qatar University, Doha, Qatar

**Keywords:** Depression, Anxiety, Body dysmorphic disorder, Body contouring surgery, Bariatric surgery

## Abstract

**Background:**

Body image reflects how individuals perceive, evaluate, and emotionally respond to their physical appearance. Massive weight loss often leads patients to seek body contouring procedures to relieve both physical discomfort and psychological distress from excess skin. While primarily esthetic, such surgeries have shown psychological benefits. This study assessed the association between body contouring and changes in psychometric scores and identified predictors of improved outcomes.

**Methods:**

Patients undergoing body contouring at Hamad General Hospital between January 2021 and December 2023 were evaluated using the Appearance Anxiety Inventory (AAI), Patient Health Questionnaire-9 (PHQ-9), and Generalized Anxiety Disorder-7 (GAD-7). Scores were categorized into a low-risk group (LRG; ≤ 2) and higher-risk group. Hormone concentrations of active ghrelin (AG), glucagon-like peptide-1 (GLP-1), leptin, spexin, and peptide YY were measured. Logistic regression, adjusted for repeated measures, examined predictors of LRG status.

**Results:**

Forty patients completed baseline assessments, with 19 completing Visit 3 (6–10 weeks postoperatively). Postoperative status was statistically divergently associated with LRG membership for PHQ-9 (a nearly fourfold increase in odds 6–10 weeks after surgery), and AAI (a nearly fivefold increase in odds 6–10 weeks after surgery). AG levels demonstrated the strongest and most statistically divergent positive association with LRG membership for GAD-7.

**Conclusion:**

Body contouring surgery is associated with short-term psychological improvements, reflected in higher odds of being in a LRG for PHQ-9, GAD-7, and AAI 6–10 weeks after surgery. The strong postoperative association with LRG status underscores the mental health benefits of surgical reshaping, suggesting that such procedures may positively influence psychological well-being although such well-being is also modulated by nutrient-stimulated hormones.

**Level of Evidence II:**

This journal requires that authors assign a level of evidence to each article. For a full description of these Evidence-Based Medicine ratings, please refer to the Table of Contents or the online Instructions to Authors www.springer.com/00266.

## Introduction

Body image refers to how individuals perceive, evaluate, and emotionally respond to their physical appearance and their behaviors based on these perceptions [1]. For some, concerns about appearance can be a passing discomfort; for others, they become deeply distressing and clinically significant. Disturbances in body image are commonly associated with psychiatric conditions such as body dysmorphic disorder (BDD), depression, anxiety, and other appearance-related disorders [2, 3]. Importantly, these psychological challenges often persist even after substantial weight loss, especially in patients who struggle with the presence of excess skin or continued dissatisfaction with their appearance. This divergence between physical change and emotional resolution underscores the complex and often underrecognized influence of body image on overall psychological well-being.

Individuals who have experienced massive weight loss often pursue body contouring procedures, such as liposuction, abdominoplasty, or augmentation mastopexy, to address both the physical burden and psychological distress associated with excess skin. Although these surgeries are primarily esthetic, increasing evidence suggests they offer substantial psychological benefits. Patients who undergo body contouring procedures frequently report reduced body image dissatisfaction [1]. In addition, patients who undergo body contouring surgery after bariatric procedures report significantly greater improvements in quality of life and psychological well-being compared to those who did not undergo such surgery [2]. These findings underscore the broader psychosocial impact of body contouring procedures and their potential role in supporting mental health recovery.

Quantitative assessments using validated psychometric tools—such as the Appearance Anxiety Inventory (AAI), Patient Health Questionnaire-9 (PHQ-9), and Generalized Anxiety Disorder-7 (GAD-7)—have begun to outline favorable outcomes after body contouring procedures [4]. These include reduction in depressive symptoms enhancing self-esteem and facilitating social reintegration. Specifically, more than half of patients who undergo abdominoplasty experience enhanced quality of life and improved self-esteem, with the vast majority indicating they would choose to undergo the procedure again [5, 6]. To extend the available data in this area, this study aims to evaluate the association between body contouring surgeries and changes in psychometric scores, as well as to identify the predictors influencing each psychometric outcome.

## Methods

### Study Population

This study included patients who underwent body contouring surgeries at the Department of Plastic Surgery, Hamad General Hospital, between January 2021 and December 2023. Participants were prospectively assessed at four time points: preoperatively (within 1 week before surgery), immediate postoperatively (2–3 weeks after surgery), intermediate postoperatively (6–10 weeks after surgery), and late postoperatively (6–10 months after surgery) to evaluate the study variables. Eligible participants were consenting adults (≥ 18 years) with a BMI ≥ 18 undergoing abdominoplasty, lower body lift, or thigh lift (thighplasty). Exclusion criteria included recent bariatric surgery (within 18 months), significant comorbidities, diabetic nephropathy, body contouring procedures outside the abdomen or thigh, age over 65 years, and BMI > 35.

### Assessment of Body Composition

The body composition of enrolled participants was evaluated at predetermined intervals (both preoperatively and postoperatively) utilizing the Tanita DC-360 P body composition analyzer. This device employs bioelectrical impedance analysis (BIA) technology to conduct comprehensive whole-body composition assessments [7].

### Ethical Approval

The study received institutional review board approval (IRB) from Hamad Medical Corporation under reference MRC-01-20-466.

### Surgical Interventions

The study participants who satisfied the eligibility criteria received either abdominoplasty, lower body lift, thigh lift procedures, or combinations thereof, all performed in accordance with established surgical protocols. These operations were conducted exclusively by plastic surgeons performing body contouring procedures at the institution.

### Psychological Assessment

Psychological status was assessed using three validated tools: AAI, PHQ-9, and GAD-7. The AAI is a 10-item questionnaire measuring appearance-related anxiety, with each item scored on a 5-point Likert scale (0–4), yielding a total score between 0 and 40; higher scores indicate greater anxiety related to body image. The PHQ-9 assesses depressive symptoms across 9 items, each scored from 0 to 3, for a maximum score of 27, with higher scores reflecting more severe depression. The GAD-7 evaluates generalized anxiety through 7 items, also scored from 0 to 3, with a total score range of 0–21; higher scores correspond to greater anxiety severity. All three assessment tools were administered at Visits 1, 3, and 4. The scores from all tools were categorized into a low-risk group (LRG; defined as a score of 2 points or below) versus everyone else, to ensure separation of low-risk rated subjects from others with higher risk of appearance-related anxiety, depressive symptoms, or generalized anxiety.

### Hormonal Assessment

Blood samples were collected in pre-chilled ethylenediaminetetraacetic acid (EDTA)-coated tubes and immediately stored at 4 °C, followed by centrifugation at 1500 × g for 10 minutes at 4 °C using a pre-cooled centrifuge to separate plasma, which was then aliquoted into 1 mL FluidX Tri-Coded cryovials and stored at − 70 °C until analysis. Hormone concentrations of active ghrelin (AG), glucagon-like peptide-1 (GLP-1), leptin, and peptide YY (PYY) were measured using EMD Millipore's MILLIPLEX® Human Metabolic Hormone Panel V3 with Luminex xMAP technology for simultaneous multi-analyte detection in various biological matrices. Spexin levels were determined using ELISA kits from Abbexa Ltd. All samples were analyzed in duplicate within a single assay, and the intra-assay coefficient of variation was kept below 10% for optimal accuracy and precision.

### Sample Size

Sample size calculations were not undertaken because they require prior knowledge of the true effect size, which is inherently unknown both before and after a study is conducted; if the true effect size was known, conducting the study would be unnecessary [8]. Post hoc power calculations were also not performed, as they are fundamentally problematic: They are often uninformative, susceptible to bias, and subject to substantial sampling variability, rendering them unreliable and inappropriate [9–12]. Instead, this study included all participants available during the study period, thereby ensuring a comprehensive and inclusive dataset.

### Statistical Analysis

Baseline characteristics were summarized using means ± standard deviations for continuous variables and counts (percentages) for categorical variables. Our primary analysis focused on the predictors of the LRG, with adjustments for repeated measures to account for multiple observations per patient over time. We employed multivariable logistic regression to examine predictors of the LRG in each tool by visit (a proxy for body countering surgery status since Visit 1 is pre-surgical and Visit 3 was 6–10 weeks post-surgery), history of bariatric surgery, and hormonal predictors. Due to lack of questionnaire data at visits 2 and 4, these were not analyzed.

To determine if the study data were consistent with sampling from a population that assumes no effect, a *p*-value was computed from the data to rank it on the hypothesized model of this population. The smaller the *p*-value, the more divergent the estimated effect is from the assumed no-effect population and results in the interval *p *< 0.05 were labeled ‘statistically divergent’ meaning that some other model of the population (other than the assumed no-effect model) is more likely to have generated the study data [13]. To assess clinical benefit, the point estimate of the population effect and its 95% uncertainty interval (95% UI; the range of potential effects in the population where the data would fall in the central 95% of the distributions specified by the model) were reported with an assessment of the practical importance of the study result. All analyses were conducted using Stata Version 17.

## Results

The study included a total of 40 participants at baseline (Visit 1), with 19 participants completing Visit 3 (6–10 weeks postoperatively). No significant differences were observed in gender distribution, age, or total body fat percentage between the two visits. Hormonal assessments showed generally low postoperative levels of leptin, spexin, GLP-1, and PYY compared to baseline, though this trend did not reach statistical divergence. However, an opposite trend was observed in AG levels, with an increase in postoperative values with no observed statistical divergence. In contrast, psychometric outcomes revealed marked improvements postoperatively. PHQ-9 scores significantly decreased from 3.95 ± 4.03 at baseline to 1.11 ± 1.45 at Visit 3 (*p* = 0.004), while GAD-7 and AAI scores showed strong divergence from the tested model (*p* = 0.058 and *p* = 0.063, respectively), suggesting that there was likely a change in psychological well-being (improvement) following body contouring procedures (Table 1).

**Table 1:** Baseline characteristics of study population

Factor	Levels/units	Visit 1	Visit 3	*P*-value
Number of participants		40	19	
Gender	Female	31 (77.50%)	15 (78.95%)	0.90
	Male	9 (22.50%)	4 (21.01%)	
Body contouring surgery type	Abdominoplasty ± LS Lower body lift ± LS Thigh lift ± LS	30 (75%) 8 (20%) 2 (5%)	15 (79%) 2 (10.5%) 2 (10.5%)	
Age, mean (SD)		41.33 (9.46)	41.67 (10.40)	0.90
Total fat %, mean (SD)		35.34 (9.38)	33.35 (8.54)	0.44
History of bariatric surgery	0 1	20 (50.00%) 20 (50.00%)	10 (52.63%) 9 (47.37%)	0.85
PYY, mean (SD)	pg/ml	38.38 (85.27)	10.07 (31.14)	0.17
Active ghrelin, mean (SD)	pg/ml	0.77 (2.75)	2.09 (6.06)	0.25
Leptin, mean (SD)	µg/L	11.94 (10.65)	11.49 (7.84)	0.87
Spexin, mean (SD)	pg/ml	411.39 (375.41)	358.20 (285.81)	0.59
GLP-1 total, mean (SD)	pg/ml	219.95 (241.71)	207.12 (165.34)	0.84
GAD-7 score, mean (SD)		3.65 (5.01)	1.26 (2.79)	0.058
PHQ-9 score, mean (SD)		3.95 (4.03)	1.11 (1.45)	0.004
AAI score, mean (SD)		6.95 (6.50)	3.68 (5.45)	0.063

*AAI* Appearance anxiety inventory; *PHQ-9* Patient health questionnaire-9; *GAD-7* Generalized anxiety disorder-7; *PYY* Peptide YY; *GLP-1* Glucagon-like peptide-1; *SD* Standard deviation; *LS* Liposuction

### LRG–GAD-7

Logistic regression analysis revealed that AG levels showed the strongest and most statistically divergent association with low GAD scores, indicating a positive association (OR = 1.45, *p* = 0.015). This suggests that higher AG levels were associated with a 45% increase in the odds of being in the low anxiety group. Additionally, Visit 3 (postoperative) had a nearly threefold increase in the odds of LRG compared to Visit 1 (OR = 2.87, *p* = 0.089), demonstrating strong statistical divergence from the tested hypothesis. Male gender, increasing levels of leptin and spexin, and decreasing levels of PYY were also associated with a higher odds of the LRG, but these associations were only weakly statistically divergent. The ROC curve had an AUC of 0.7723, indicating moderate discriminative ability of the model in classifying the outcome of interest. Overall, the strongest associations with LRG–GAD were observed post-body contouring surgery and with higher AG levels (Table 2).

**Table 2:** Predictors of LRG-GAD-7 using logistic regression*

LRG-GAD-7	Odds ratio (OR)	*P*-value	95% Uncertainty Interval
*Visit*			
1	1		
3	2.87	0.089	0.852–9.673
*Gender*			
Female	1		
Male	4.16	0.141	0.624–27.72
PYY	0.994	0.121	0.987–1.001
Active ghrelin	1.5	**0.015**	1.07–1.957
Leptin	1.00	0.241	0.99995–1.0002
Spexin	1.0015	0.210	0.9992–1.004
Constant	0.39	0.263	0.0735–2.042

*GAD-7* Generalized anxiety disorder-7; *LRG* Low-risk group; *PYY* Peptide YY

^*^Goodness of fit: AUC 0.7723

Bold value indicate variables showing greater statistical divergence from the no-effect model in the regression analysis at the predefined threshold

### LRG–PHQ-9

Logistic regression analysis revealed that postoperative status was significantly associated with the LRG, with a nearly fourfold increase in LRG membership at Visit 3 compared to Visit 1 (OR = 3.97, *p* = 0.002). PYY levels demonstrated an inverse trend with the LRG (OR = 0.993, *p* = 0.070), suggesting that lower PYY levels may be linked to the LRG, with strong statistical divergence. Other variables, including bariatric surgery status (OR = 1.66, *p* = 0.326), AG levels (OR = 1.12, *p* = 0.294), and total GLP-1 (OR = 1.003, *p* = 0.111), showed weakly statistically divergent associations with the LRG though all demonstrated increased odds of the LRG. The ROC curve analysis yielded an AUC of 0.7168. Overall, the only highly statistically divergent predictor of improved depressive status was having had body contouring surgery (Table 3).

**Table 3:** Predictors of LRG-PHQ-9 using logistic regression*

LRG-PHQ-9	Odds ratio (OR)	*P*-value	95% Uncertainty interval
*Visit*			
1	1		
3	3.97	**0.002**	1.693–9.288
*History of bariatric* surgery			
No	1		
Yes	1.67	0.326	0.603–4.59
PYY	0.993	0.070	0.986–1.0005
Active ghrelin	1.12	0.294	0.91–1.389
GLP-1 total	1.00	0.111	0.9995–1.01
Constant	0.49	0.126	0.1924–1.225

*PHQ-9* Patient health questionnaire-9; *LRG* Low-risk group; *PYY* Peptide YY; *GLP-1* Glucagon-like peptide-1

^*^Goodness of fit: AUC 0.7168

Bold value indicate variables showing greater statistical divergence from the no-effect model in the regression analysis at the predefined threshold

### LRG–AAI

Logistic regression analysis revealed that there was a very strongly statistically divergent association with LRG, demonstrating a nearly fivefold increase in the odds of LRG post-compared to pre-body contouring surgery (OR = 4.72, *p* = 0.006). Spexin levels also demonstrated a statistically divergent positive association with LRG (OR = 1.0016, *p* = 0.025), suggesting that higher spexin concentrations may be linked to reduced appearance anxiety. In contrast, bariatric surgery status showed very weakly divergent associations, as did leptin and total GLP-1 levels. The ROC curve analysis yielded an AUC of 0.7317. Among all variables, postoperative status and spexin were the only strongly statistically divergent predictors of LRG related to appearance-related anxiety (Table 4).

**Table 4:** Predictors of LRG-AAI using logistic regression*

LRG - AAI	Odds ratio (OR)	*P*-value	95% Uncertainty Interval
*Visit*			
1	1		
3	4.72	**0.006**	1.56–14.3
*History of bariatric surgery*			
No	1		
Yes	0.782	0.662	0.259–2.4
Leptin	1.000	0.194	0.99998–1.0001
Spexin	1.002	**0.025**	1.0002–1.003
GLP-1 total	0.999	0.367	0.996–1.002
Constant	0.25	0.048	0.06–0.999

*AAI* Appearance anxiety inventory; *LRG* Low-risk group; *GLP-1* Glucagon-like peptide-1

^*^Goodness of fit: AUC 0.7317

Bold values indicate variables showing greater statistical divergence from the no-effect model in the regression analysis at the predefined threshold

## Discussion

Our study assessed the psychological impact of body contouring surgery and examined both surgical and hormonal predictors of the LRG. We found that being 6–10 weeks post-surgery was the strongest predictor of belonging to the LRG, defined by PHQ-9, GAD-7, or AAI scores of less than 3 on their respective scales. The odds of LRG across all three scales significantly increased after surgery, supporting a positive effect of body contouring on mental well-being.

These findings align with some previous studies reporting improvements in psychological health, including reductions in anxiety and depression, after body contouring procedures [4, 14–17]. Specifically, abdominoplasty has been associated with enhanced self-esteem and reduced psychological distress [14, 15]. However, some studies have found no significant change in anxiety or depression postoperatively, though they still observed gains in body image and self-acceptance [2, 18]. It is therefore possible that the benefit seen within 6–10 weeks of surgery in this study may diminish over time. Importantly, our analysis also identified nutrient-stimulated hormones as independent predictors of psychological outcomes, even after accounting for surgical status. Among these, AG showed the strongest association with LRG for GAD-7, suggesting that higher AG levels are linked to reduced anxiety. This is consistent with animal studies where exogenous ghrelin reduced anxiety-like behaviors following stress exposure [19–21]. Human data remain inconsistent—some studies report a dual role for ghrelin depending on the anxiety subtype [22], while others found no association with anxiety or depression [23, 24], although several have suggested correlations with depressive symptoms [25, 26]. PYY was inversely associated with odds of LRG for PHQ-9 in our study, suggesting that reduced PYY may be linked to improved mood. A weakly statistically divergent association was also observed for LRG for GAD-7. While evidence in humans is limited, animal studies have demonstrated anxiolytic and antidepressant-like effects of PYY [27, 28]. However, no clear association has been found in obese human populations [24]. Similarly, GLP-1 showed a weakly divergent association with LRG for AAI and PHQ-9. While direct human data are lacking, GLP-1 receptor agonists (GLP-1RAs) have shown potential psychiatric benefits in patients with type 2 diabetes, including reduced healthcare utilization for mood disorders and protective effects against anxiety [29–31]. However, a Mendelian randomization study found insufficient evidence for a causal relationship between GLP-1RAs and anxiety or depression [32].

We also studied two adipokines and the first one, leptin, was not statistically divergent in its association with any of the psychometric outcomes in our study. The literature presents mixed findings: Some studies link elevated leptin to increased anxiety and depression, especially in men with obesity [23, 25, 33], while others report the opposite, associating lower leptin levels with mood disorders in women and individuals with body dysmorphic disorder [34, 35]. These inconsistencies suggest that the effects of leptin on mental health may be influenced by other factors such as sex, body composition, and possibly other hormonal or behavioral mediators [36–38]. We also observed an association between spexin and LRG for AAI, but not for GAD-7. Although human data are scarce and inconclusive [39], animal models suggest that spexin may have regulatory effects on mood [40].

Although the study demonstrated several positive outcomes—including integrated psychometric and endocrine assessment showing statistically divergent short-term improvements in PHQ-9, GAD-7, and AAI scores, and identifying nutrient-stimulated hormones, particularly acyl-ghrelin and peptide YY, as independent predictors of improved outcomes—the high attrition rate reduced the follow-up sample size. Accordingly, these findings should be interpreted as associative and hypothesis-generating, and larger, well-powered studies are needed for validation. In addition, complications that may impact psychological well-being were not assessed. However, our group has recently published data evaluating complication rates following liposuction alone, demonstrating that overall complication rates are low, generally remaining below 2% [41]. Even assuming that body contouring procedures may carry complication rates approximately twice those observed with liposuction, the expected overall rate would still remain below 4%. Given the relatively low incidence of postoperative complications, it is unlikely that such events would materially influence the psychological outcomes observed following body contouring surgery.

## Conclusion

Our findings clearly support the role of body contouring surgery in improving short-term psychological well-being, with a statistically divergent increase in the odds of LRG for PHQ-9, GAD-7, and AAI. The strong association between LRG status and the postoperative period highlights the positive mental health impact of surgical reshaping. Furthermore, our identification of nutrient-stimulated hormones—particularly AG and PYY—as independent predictors of improved outcomes adds a novel dimension to the understanding of mood regulation in this context. These results underscore the importance of integrating psychometric evaluation into post-surgical care and suggest a potential for the future study of biological pathways linking metabolic and emotional recovery.
